# In Vitro and In Vivo Evaluation of Commercially Available Fibrin Gel as a Carrier of Alendronate for Bone Tissue Engineering

**DOI:** 10.1155/2017/6434169

**Published:** 2017-01-22

**Authors:** Beom Su Kim, Feride Shkembi, Jun Lee

**Affiliations:** ^1^Bonecell Biotech. Inc., Dunsan-dong, Seo-gu, Daejeon 602-830, Republic of Korea; ^2^Wonkwang Bone Regeneration Institute, Wonkwang University, Iksan 570-749, Republic of Korea; ^3^Department of Oral & Maxillofacial and Surgery, Wonkwang University Daejeon Dental Hospital, Seo-gu, Daejeon 302-120, Republic of Korea

## Abstract

Alendronate (ALN) is a bisphosphonate drug that is widely used for the treatment of osteoporosis. Furthermore, local delivery of ALN has the potential to improve the bone regeneration. This study was designed to investigate an ALN-containing fibrin (fibrin/ALN) gel and evaluate the effect of this gel on both in vitro cellular behavior using human mesenchymal stem cells (hMSCs) and in vivo bone regenerative capacity. Fibrin hydrogels were fabricated using various ALN concentrations (10^−7^–10^−4 ^M) with fibrin glue and the morphology, mechanical properties, and ALN release kinetics were characterized. Proliferation and osteogenic differentiation of and cytotoxicity in fibrin/ALN gel-embedded hMSCs were examined. In vivo bone formation was evaluated using a rabbit calvarial defect model. The fabricated fibrin/ALN gel was transparent with Young's modulus of ~13 kPa, and these properties were not affected by ALN concentration. The in vitro studies showed sustained release of ALN from the fibrin gel and revealed that hMSCs cultured in fibrin/ALN gel showed significantly increased proliferation and osteogenic differentiation. In addition, microcomputed tomography and histological analysis revealed that the newly formed bone was significantly enhanced by implantation of fibrin/ALN gel in a calvarial defect model. These results suggest that fibrin/ALN has the potential to improve bone regeneration.

## 1. Introduction

Bone defects occur as a result of various conditions such as tumors, trauma, disease, and fracture. Although small defects have the capacity to self-regenerate, large-sized defects do not heal well. To enhance bone repair and reconstruction, the use of osteogenic growth factors or cytokines such as bone morphogenetic protein-2 (BMP-2) has been explored [1, 2].

Recently, several studies have suggested that alendronate (ALN), one of the bisphosphonates (BPs), can enhance the osteogenesis of osteoblasts and mesenchymal stem cells (MSCs) [3–5]. BPs are well known potential antiresorptive drugs and are widely used in the treatment of metabolic bone diseases [6]. BPs are classified into two main groups: nonnitrogen-containing BPs, which produce nonhydrolyzable analogs of ATP that are toxic to cells, and nitrogen-containing BPs, which are more potent drugs that inhibit the mevalonate pathway and therefore indirectly induce osteoclast apoptosis [7, 8].

Among the BPs, alendronate (ALN) is one of the most potent nitrogen-containing BPs and is commonly used for treating osteoporosis [9]. The mechanism of ALN is attributed to the inhibition of osteoclast precursor cells through suppression of farnesyl diphosphate synthase, a key enzyme of the mevalonate pathway [10]. A previous study reported that ALN stimulates the proliferation and osteoblast differentiation of human bone marrow-derived MSCs [3]. Furthermore, Toker et al. reported that locally implanted ALN promoted bone formation in a rat critical-sized calvarial defect model [11]. However, ALN easily dissolves in aqueous conditions because of its high hydrophilicity, and uncontrolled burst release can lead to side effects. Therefore, to achieve efficient osteogenesis, proper ALN delivery carriers are required for implantation at bone defect sites.

Fibrin gel consists of fibrinogen and thrombin components isolated from human plasma. When fibrinogen is activated by thrombin, it is converted into a fibrin monomeric biopolymer and forms a fibrin gel [12]. The fibrin gel is frequently used as a drug delivery system [13] or scaffold material [14] because it is biocompatible and naturally biodegrades, and it is possible to vary the release profile from hours to weeks [15]. Furthermore, fibrin promotes osteoblast differentiation and accelerates new bone formation from osteoblasts located near the bone membrane [16, 17]. However, the biocompatibility and in vivo bone healing properties of an ALN-loaded fibrin gel system have not been sufficiently studied.

In this study, we fabricated an ALN-loaded fibrin gel and the mechanical properties and release pattern were characterized. The biocompatibility was evaluated using human MSCs in vitro. The in vivo bone formation capacity of the fibrin/ALN gel was evaluated using a rabbit calvarial defect model.

## 2. Materials and Methods

### 2.1. Preparation ALN-Containing Fibrin Gel

**Figure 1:**
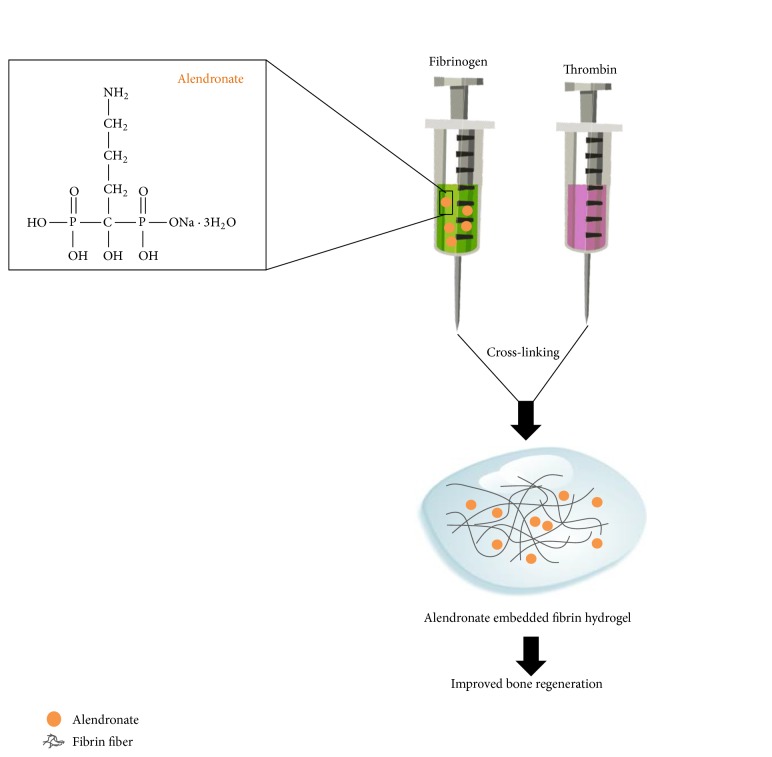
Schematic diagrams of the alendronate embedded fibrin hydrogel to improve bone regeneration.

To fabricate ALN-containing fibrin gel, ALN dissolved fibrinogen solution was polymerized using thrombin.  Figure 1 shows a schematic diagram of preparation the ALN embedded fibrin gel to improve bone regeneration. Briefly, ALN was dissolved in phosphate buffered saline (PBS) at various concentrations (10^−7^–10^−4 ^M). Fibrinogen powder (Greenplast; Greencross, Seoul, Korea) was dissolved in the prepared ALN solution and poured into a silicon mold. To induce polymerization, thrombin solution was added and blended. The composite mixture was polymerized at 37°C for 10 min. Fibrin gels were constructed so that the final concentrations were 20 mg/mL fibrinogen and 2 IU/mL thrombin.

### 2.2. Mechanical Strength

We measured Young's moduli of fibrin/ALN gel constructs prepared with various concentrations of ALN. The fabricated fibrin/ALN gels were loaded between two flat plates and compressed at a rate of 0.5 mm/min using an Instron 3345 (Norwood, MA, USA). Young's moduli were determined based on the 0–5% linear region of the stress-strain curve [18].

### 2.3. In Vitro ALN Release

The fibrin/ALN gel samples were soaked in 1 mL PBS (pH 7.4). At predetermined time intervals, 0.1 mL of the sample media was collected and replaced with fresh solution. ALN concentration was determined spectrophotometrically at 293 nm via complex formation with Fe(III) ions, as previously described [19].

### 2.4. Cell Culture

To evaluate biocompatibility, alveolar bone marrow-derived MSCs obtained from Professor You were utilized (Wonkwang University, Iksan, Korea). The cells were cultured in *α*-MEM (Invitrogen, Carlsbad, CA) containing 10% fetal bovine serum (FBS; Invitrogen) and 1% penicillin/streptomycin at 37°C, 5% CO_2_, and 100% humidity. The hMSCs were passaged 3–6 times.

### 2.5. Preparation of Cells Embedded in ALN-Loaded Fibrin Gel Constructs

Human mesenchymal stem cells (hMSCs) were dissociated using a 0.05% trypsin/EDTA solution. An aliquot containing 2 × 10^4^ cells was resuspended in 100 *μ*L of fibrinogen only or ALN-containing fibrinogen solutions. The suspensions were then poured into silicone molds. Polymerization was induced by adding thrombin solution (20 *μ*L, 2 IU/mL) with mixing. The constructs were briefly washed with PBS to remove residual thrombin and the cell-containing gels were placed into 24-well plates and cultured as described above.

### 2.6. Cell Proliferation Assay

The proliferation of cells embedded in fibrin/ALN gel was analyzed using CellTiter96® Aqueous One solution (Promega, Madison, WI). Briefly, after 3 days of cultivation, 50 *μ*L MTS reagent was added to each well and incubated for 2 h. Then, 100 *μ*L supernatant was removed and the absorbance at 490 nm was measured using a SpectraMAX M3 plate reader (Molecular Devices, Sunnyvale, CA).

### 2.7. Viability and Cytotoxicity Assay

After 3 days of cultivation, hMSC-embedded gels were soaked in Dulbecco's phosphate buffered saline (DPBS) for 30 min to remove phenol red and serum. Staining was performed in dark conditions for 30 min using a Live/Dead® Viability/Cytotoxicity kit (Molecular Probes, UK). Calcein acetoxymethyl (calcein AM, 0.05%) stains viable cells green and ethidium homodimer-1 (EthD-1, 0.2%) stains the nuclei of nonviable cells red. The stained samples were examined under an inverted fluorescence microscope (DM IL LED Fluo; Leica Microsystems, Wetzlar, Germany).

### 2.8. Alkaline Phosphatase (ALP) Activity

ALP activity was measured using a* p*-nitrophenyl phosphate-based colorimetric assay. Briefly, the cell-embedded gels were cultured each gel. After 7 days of cultivation, gel constructs were homogenized in 100 *μ*L Tris (pH 8.0) buffer and lysed via ultrasonication for 5 min. The supernatant was then collected for the ALP activity assay using* p*-nitrophenylphosphate (*p*-NPP) as a substrate, as described previously [20].

### 2.9. Calcium Accumulation Assay

Calcium accumulation was assayed by alizarin red S staining. Embedded cells were cultured for 2 weeks during which medium was replaced every two days. The gel encapsulated cells were fixed with ice-cold 70% ethanol for one hour and ethanol was removed. Next, alizarin red S staining solution (40 mM, pH 4.2) was added and the cells were incubated for 30 min. The stained samples were washed five times for 30 min with distilled water and stained portions were photographed with a digital camera (EOS 500D; Canon, Tokyo, Japan). Quantification of calcium accumulation was carried out by chopping hydrogel samples into small pieces, and stained portions were solubilized using 10% (w/v) cetylpyridinium chloride in 10 mM sodium phosphate buffer (pH 7.0) and the absorbance measured at 562 nm.

### 2.10. Real-Time Polymerase Chain Reaction

After 7 days of cultivation, total mRNA was extracted from cells embedded in fibrin gel using an RNA isolation kit (Ribospin, GeneAll, Seoul, Korea) according to the manufacturer's instructions. PCR was performed using a TaqMan Universal PCR Master Mix, TaqMan primers, and probe sets (Applied Biosystems, Carlsbad, CA, USA) specifically targeting collagen type I (*Col1AI*; Hs00164004_m1), runt related transcription factor (*Runx2;* Hs00231692_m1), osteocalcin (*OCN; *Hs01587814_g1), and* 18S* (Hs99999901_s1). The* 18S *rRNA gene was used as an internal standard. The relative expression was normalized to the control group.

### 2.11. Animal Experiment

In order to test the bone regeneration capability of fibrin/ALN gel in an animal model, fibrin gel and fibrin/ALN gel were polymerized with thrombin solution at a final concentration of 20 mg/mL fibrinogen and 10^−6 ^M ALN as described above. The experimental procedures were approved by the Institute of Laboratory Animal Research, Wonkwang University. New Zealand white rabbits, aged 3 months and weighing approximately 2.5–3.0 kg, were used in the study. All the animals were anesthetized by intramuscular injection with a combination of 35 mg/kg ketamine (Yuhan ketamine®, Yuhan Crop., Seoul, Korea) and xylazine (Celactal®, Bayer Animal Health Crop., Seoul, Korea) and local anesthesia on the surgical site using 2% lidocaine solution. We approached the calvaria via linear incision through the skin and subcutaneous tissue over the medial line and induced two separate circular defects using a trephine bur with an outer diameter of 8 mm. The animal study was performed using four rabbits at each time-point in all experiments with two defects induced in each rabbit. One of the calvarial defects was filled with fibrin gel without ALN and the other was filled with fibrin/ALN gel.

At 2, 4, and 8 weeks after surgery, bone tissue defects were removed from the host bone and fixed with 4% paraformaldehyde buffer (pH 7.2) before performing further experiments.

### 2.12. Microcomputed Tomography (CT) Evaluation

In order to evaluate the volume of regenerated bone, both defects were analyzed using three-dimensional micro-CT (Sky-Scan 1172™; Skyscan, Kontich, Belgium). The medium resolution was 8.82 *μ*m and the X-ray generator was on an operating voltage of 60 kV with a constant current of 167 *μ*A. The beam was filtered through a 0.5 mm aluminum filter. The image data were reconstructed and three-dimensional images were obtained. Based on the micro-CT data sets, the newly formed bone volume was calculated within the region of interest.

### 2.13. Histological Analysis

After micro-CT scanning, all specimens were decalcified in 10% EDTA and then dehydrated in a graded alcohol series ranging from 70 to 100%. Paraffin blocks were prepared using a routine process and were cut into 5 *μ*m sections (HM 325; Microm, Walldorf, Germany). For each sample, we stained five sections with hematoxylin & eosin (H&E) and Goldner's Masson trichrome, using standard techniques.

### 2.14. Statistical Analysis

All experiments were performed in triplicate. Values are expressed as mean ± standard deviation (SD) and one-way analysis of variance (ANOVA) followed by a post *t*-test was performed using GraphPad Prism version 5.3 (GraphPad Software, SanDiego, CA, USA). *P* < 0.05 was considered statistically significant.

## 3. Results

### 3.1. Morphological/Mechanical/Release Characterization of Fibrin/ALN Gels

**Figure 2:**
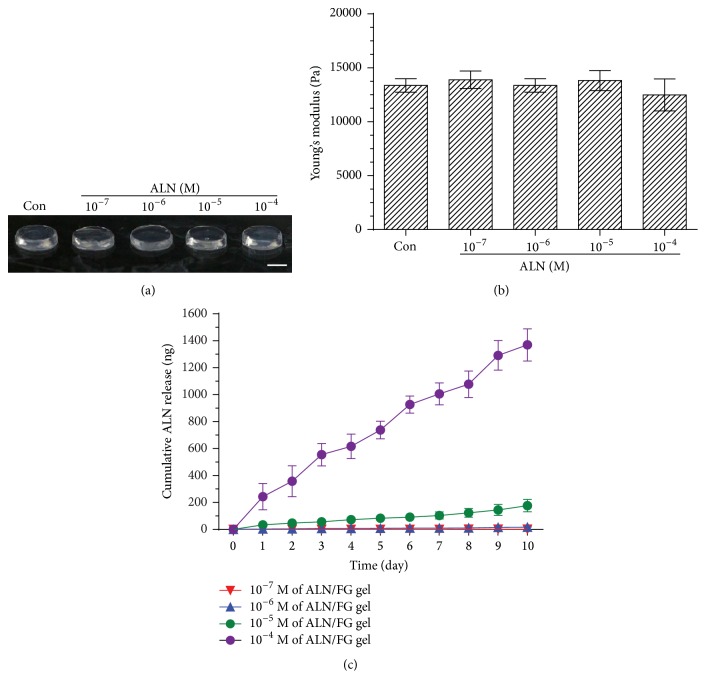
(a) Gross view of fibrin gels containing various ALN concentrations (10^−4 ^M~10^−7 ^M). (b) Effects of ALN concentration on the stiffness of fibrin/ALN gel. The stiffness of the fibrin/ALN gel was not significantly affected by ALN concentration. (c) In vitro cumulative release of ALN from fibrin gel. A slow sustained release was observed. The data shown are the mean ± standard deviation (SD) of three independent experiments.

Fibrin/ALN gels were formulated with various concentrations of ALN. The formed gels were solid-like, semitransparent in appearance, and not affected by ALN concentration (Figure 2(a)). Next, we measured the compressive moduli of the fibrin/ALN gels. Young's modulus of the gels prepared with fibrin alone was 13.37 ± 0.6 kPa and did not change significantly with the addition of ALN (*P* > 0.05) (Figure 2(b)).

The ALN release kinetics results showed that the amount of ALN released increased with ALN concentration. Overall, the results showed a sustained ALN release of approximately 45% up until 10 days (Figure 2(c)).

### 3.2. Cell Proliferation and Cellular Response

**Figure 3:**
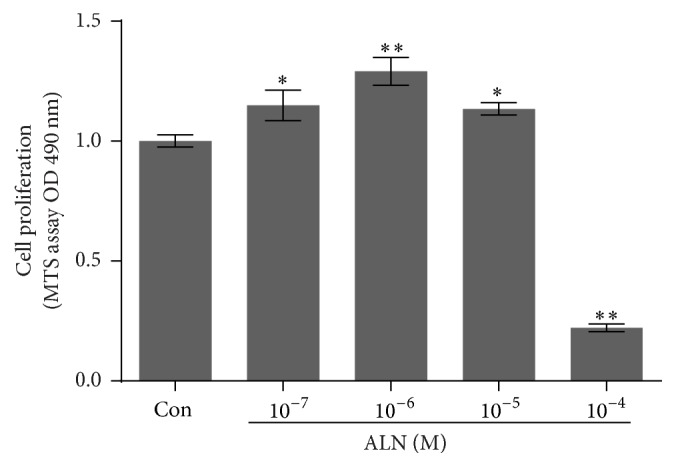
Effect of ALN concentration in fibrin/ALN gels on human mesenchymal stem cell (hMSC) proliferation. Cells were embedded and cultured in the fibrin gels prepared with ALN at various concentrations. After 3 days of cultivation, cell proliferation was measured. The cells embedded in the fibrin/ALN gels prepared with 10^−7^, 10^−6^, and 10^−5 ^M ALN exhibited increased proliferation. However, proliferation was significantly inhibited in fibrin/ALN gels prepared with 10^−4 ^M ALN. The data shown are the mean ± standard deviation (SD) of three independent experiments. ^*∗*^*P* < 0.05 and ^*∗∗*^*P* < 0.01 compared with the control.

**Figure 4:**
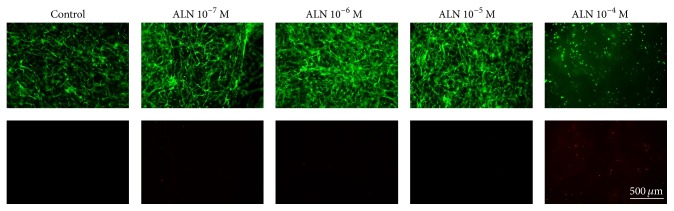
Effect of ALN concentration in fibrin/ALN gels on cell viability. Human mesenchymal stem cells (hMSCs) were embedded in fibrin/ALN gels containing various concentrations of ALN. After 3 days of cultivation, live/dead staining was performed. Most of the hMSCs were viable and retained a fibroblast-like morphology (stained by calcein AM, shown in green) in fibrin gels and 10^−7^, 10^−6^, and 10^−5 ^M ALN composite fibrin gels. In contrast, when cells were embedded in 10^−4 ^M ALN fibrin/ALN gel, a large number of cells were found to be dead or unhealthy (stained by EthD-1, shown in red) and cells were poorly distributed and had a rounded cellular morphology.

To evaluate the effect of ALN concentration on hMSCs, cells were embedded in the gel and cultured. As shown in Figure 2, cell proliferation significantly increased in 10^−6 ^M ALN gels compared with the control. The cell proliferation markedly decreased in 10^−4 ^M ALN gels (Figure 3). To assess the in vitro cytocompatibility of the fibrin/ALN gels with varying amounts of ALN, hMSCs cultured for 3 days within the gels were evaluated. The embedded cells were stained using live/dead fluorescence staining reagents and examined using a fluorescence microscope.  Figure 4 shows that most of the hMSCs were viable and retained a fibroblast-like morphology in fibrin gels and 10^−7^, 10^−6^, and 10^−5 ^M ALN composite fibrin gels. In contrast, when cells were embedded in 10^−4 ^M ALN fibrin/ALN gels, a large number of cells were found to be dead or unhealthy.

### 3.3. Osteoblast Differentiation

**Figure 5:**
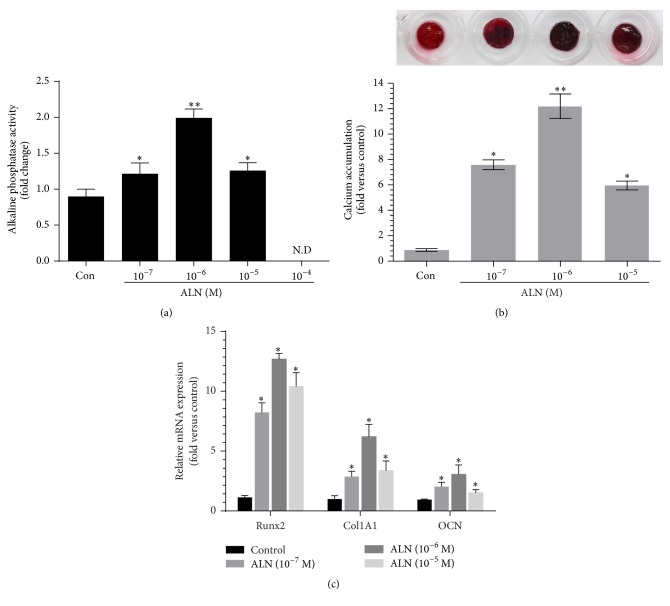
Effect of ALN concentration in fibrin/ALN gels (a) on alkaline phosphatase (ALP) activity (ND, not detected) and (b) calcium accumulation. Human mesenchymal stem cells (hMSCs) were embedded in the fibrin/ALN gels containing various concentrations of ALN. ALP activity assay and alizarin red S staining were performed after 7 and 14 days of cultivation, respectively. (c) In addition, after 7 days of cultivation, real-time PCR was performed for several osteoblast marker genes and cells cultured in fibrin/ALN gels containing 10^−7^, 10^−6^, and 10^−5^ M ALN, and osteoblast differentiation was significantly increased. The data shown are the means ± standard deviation (SD) of three independent experiments. ^*∗*^*P* < 0.05 and ^*∗∗*^*P* < 0.01 compared with control.

To determine the effect of ALN concentration of fibrin gels on osteoblast differentiation of hMSCs, ALP activity was analyzed at 7 days. Cells cultured in 10^−7 ^M, 10^−6 ^M, and 10^−5 ^M ALN fibrin gels exhibited significantly increased ALP activity compared with the control. ALP activity was not detected in 10^−4 ^M ALN fibrin gels (Figure 5(a)). In addition, calcium accumulation was analyzed to determine osteoblast differentiation. When cultured in 10^−7^, 10^−6^, and 10^−5 ^M ALN fibrin gels, the results showed significantly increased calcium accumulation with the highest accumulation detected in the 10^−6 ^M ALN fibrin gel (Figure 5(b)). Real-time PCR for several osteoblast marker genes was also used to assess osteoblast differentiation. The mRNA expression of* Col1A1*,* Runx2*, and* OCN* was significantly higher in ALN fibrin gels than in fibrin only gel (Figure 5(c)).

### 3.4. Micro-CT Evaluation

**Figure 6:**
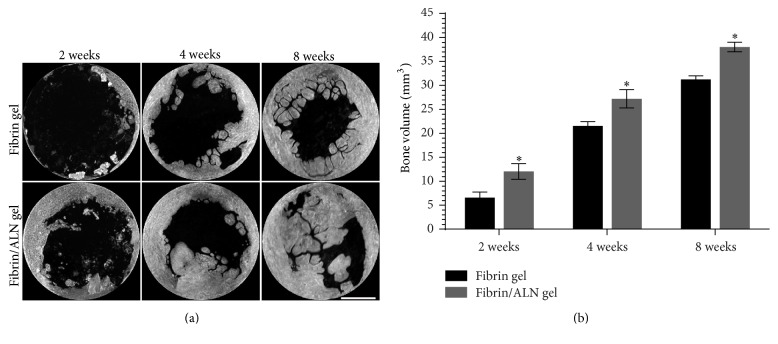
Effect of fibrin/ALN gel on new bone formation in a rabbit calvarial defect. Representative three-dimensional microcomputer tomography images (a) and quantification graph (b) of calvarial bone defect regeneration at 2, 4, and 8 weeks after implantation with fibrin gel or fibrin/ALN gel. The new bone formation increased in the fibrin/ALN gel (10^−6 ^M ALN) group compared to that in the fibrin gel-implanted group. Scale bar = 3 mm. The data shown are the mean ± standard deviation (SD). ^*∗*^*P* < 0.05 compared with the fibrin gel-implanted group.

To examine the capacity of the fibrin/ALN gels to promote bone regeneration, fibrin and fibrin/ALN gels formulated with 10^−6 ^M ALN were implanted in a rabbit calvarial bone defect. At 2, 4, and 8 weeks after implantation, the tissue was harvested and new bone formation was analyzed using micro-CT.  Figure 6(a) shows the three-dimensional images of the implanted fibrin or fibrin/ALN gels. In all groups, new bone formation occurred from the marginal defect site and progressed over time. Furthermore, 4 or 8 weeks after implantation, newly formed bone was greater in the fibrin/ALN gel-implanted group than in the fibrin gel implant group. Quantification of bone regeneration revealed significant differences in each group. Specifically, the newly formed bone volume was greater in the fibrin/ALN gel-implanted group (38.02 ± 1.00 mm^3^) than in the fibrin gel group (31.24 ± 0.76 mm^3^) at 8 weeks after implantation (Figure 6(b)).

### 3.5. Histology

**Figure 7:**
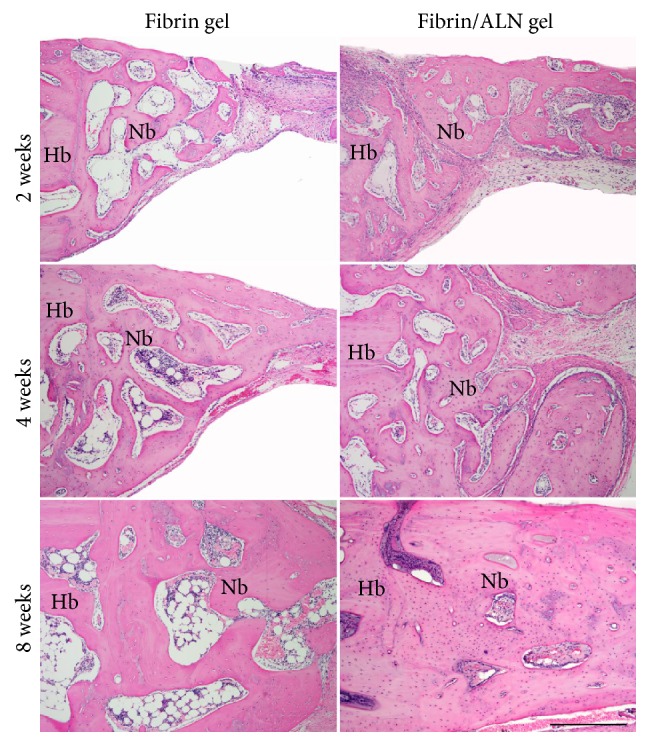
Hematoxylin and eosin-stained microscopic images of the margin of the defect site at 2, 4, and 8 weeks after implantation of fibrin gel or fibrin/ALN gel (containing 10^−6 ^M ALN). Newly formed bone at the periphery of the defect was observed in both the fibrin gel and fibrin/ALN groups from 2 weeks after implantation. Mature bone was more abundant in the fibrin/ALN group and newly formed bone tended to coalesce with the host bone. Hb, host bone; Nb, new bone. Scale bar: 250 *μ*m.

**Figure 8:**
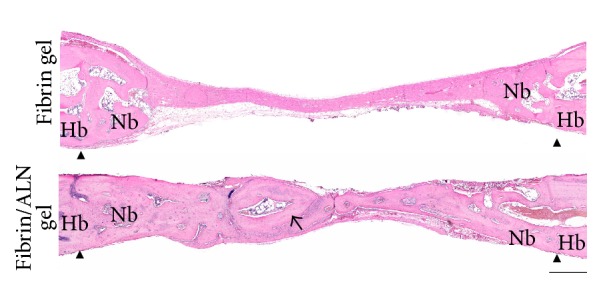
Histologic morphology of the new bone at 8 weeks after implantation. In the central area, newly formed bone was more mature in the fibrin/ALN gel-implanted group than in the fibrin gel group. The arrow indicates the newly formed bone containing woven bone and lamella. Arrow head indicates the defect marginal site. Hb, host bone; Nb, new bone. Scale bar: 500 *μ*m.

**Figure 9:**
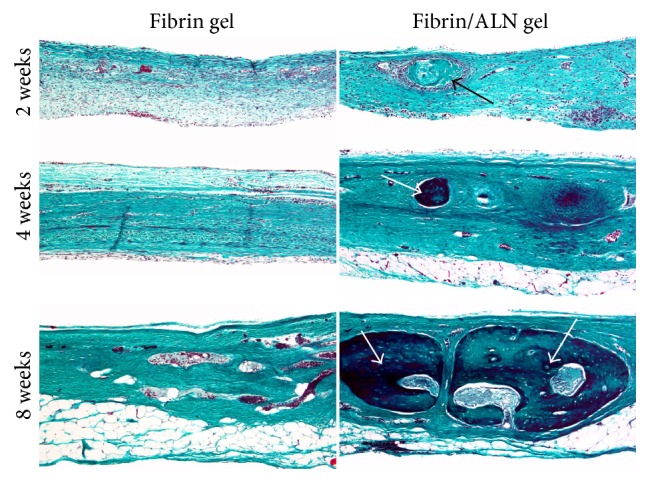
Goldner's Masson trichrome-stained histological images of regenerated bone in the central area of calvarial defects at 2, 4, and 8 weeks after implantation. At 2 weeks, large amounts of fibroblastic connective tissue were observed in the fibrin gel and fibrin/ALN gel-implanted groups. Specifically, a small island-like amount of immature bone (black arrow) was observed in the fibrin/ALN gel-implanted group. Furthermore, mature bone islands (white arrow) were observed at 4 weeks and the newly formed bones were more abundant at 8 weeks in the fibrin/ALN-implanted group than in the fibrin gel-implanted group. Scale bar: 250 *μ*m.

Histological evaluation of the fibrin/ALN gel-repaired defect specimens was performed at 2, 4, and 8 weeks after implantation.  Figure 7 shows the H&E-stained marginal site of the bone defect. Two weeks after implantation, a small amount of newly formed bone was observed at the margin of the defect in specimens implanted with fibrin gel. Otherwise, more thickness newly formed bone was observed in fibrin/ALN gel-implanted specimens. Four weeks after implantation, matured new bone was observed and newly formed bone was integrated with the host bone at the marginal aspect of the defect. At 8 weeks after implantation, the matured new bone was found to be more developed, and bone marrow cavity-like morphology was observed. Bone growth was markedly thicker in the fibrin/ALN gel group than in the fibrin gel group (Figure 8). In addition, Figure 9 shows Goldner's Masson trichrome staining of the central area of the calvarial defects. At 2 weeks after implantation, large amounts of fibroblastic connective tissue were observed in all groups. A small amount of immature bone was observed in the fibrin/ALN gel-implanted group. Furthermore, mature bone islands were observed at 4 weeks and this newly formed bone was more abundant at 8 weeks after implantation in the fibrin/ALN-implanted group. The results of the histological observation confirmed the results obtained from micro-CT analysis.

## 4. Discussion

Patients with severe bone loss could greatly benefit from tissue regeneration in large defects. Therefore, a number of studies have attempted to enhance bone regeneration using growth factors or osteoinductive drugs. Several studies have reported enhanced bone regeneration using locally applied ALN. However, the use of ALN in combination with fibrin gel for bone tissue engineering has not yet been evaluated.

In this study, we prepared ALN-containing fibrin gels and the properties of fibrin/ALN gels, including compressive moduli, ALN release kinetics, in vitro cellular behavior of hMSCs, and in vivo bone regeneration capacity were evaluated. The concentrations of fibrinogen and thrombin affect the stiffness of fibrin gels [21]. In addition, the stiffness is affected by composite materials such as protein [22]_,_ and the mechanical strength of a gel influences cellular proliferation and spreading [23]. In this study, fibrinogen (20 mg/mL) and thrombin (2 IU/mL) were used because these concentrations were optimized in pilot experiments [24], and our results showed that the stiffness was not significantly affected by ALN addition. A previous study reported that the stiffness of fibrin gel was influenced by the embedded cell density [25]. According to Jansen et al.'s report, stiffness was also affected by fibrin concentration, and significant changes only occurred at fibrin concentrations < 2 mg/mL. In keeping with these reports, in the present study, gel stiffness was not significantly changed by cell embedding (data not shown), which is likely due to the fibrinogen concentration used in this study. The fibrin gels prepared in this study were shown to contain mostly viable cells that displayed an elongated fibroblastic-like morphology.

Several researchers have investigated the local application of ALN for bone repair. However, when using local injection, drug release pattern is an important factor. According to Jeon et al., the growth factor release pattern is influenced by the fibrinogen and thrombin concentrations. They showed that when using 94.3 mg/mL fibrinogen and 33.3 IU/mL thrombin, approximately 60% of the growth factor was released after 3 days. In contrast, we observed a slow release profile and approximately 45% of the ALN was released after 10 days. The different release profiles may be the result of different concentrations of fibrinogen and thrombin [26], differences in the chemical structure and properties of the loaded drug, and preparation method [27].

In vitro cellular evaluation was the starting point for determining the biocompatibility of the formulated gel system. In this study, we first assessed cell proliferation and cytotoxicity to evaluate biocompatibility. A number of studies have reported that ALN regulates cell proliferation and differentiation [3, 28]. A previous study reported that ALN enhanced proliferation of human bone marrow stromal cells and also initiated osteoblastic differentiation involving osteogenesis-related genes such as BMP-2, bone sialoprotein-II, and Col1A1 [3]. Im et al. reported that the proliferation of MG-63 cells increased over a wide concentration range from 10^−6 ^M to 10^−9 ^M of ALN, while at 10^−4 ^M, cell proliferation decreased [5]. We observed increased cell proliferation from 10^−5^ to 10^−7 ^M and the highest concentration of 10^−4 ^M ALN inhibited cell proliferation. This is in agreement with the results reported by Im et al. [5]. This effect may be attributable to cytotoxicity of ALN at high concentrations and this is also supported by our live/dead assay results. Furthermore, they also showed that ALP activity level increased after treatment with 10^−8 ^M ALN [5]. We used ALP activity and calcium accumulation as an indicator of osteoblast differentiation. Our data showed that the ALP activity and calcium accumulation in fibrin gels increased with increasing ALN concentration from 10^−5 ^M to 10^−7 ^M and the highest osteoblast differentiation was observed in 10^−6 ^M ALN-containing fibrin gels. In 10^−4 ^M ALN fibrin gels, ALP activity was not detected because the high concentration inhibited cell proliferation, as previously described. During osteoblast differentiation,* Col1A1* [29, 30] and* Runx2* are important genes [31] that, together with* OCN,* regulate osteoblast differentiation [32]; these genes are well known osteoblast markers. Therefore, to confirm ALN-induced osteoblast differentiation, we performed real-time PCR for these marker genes and showed that their induction was related to ALN concentration in the fibrin gel system. The results suggest that fibrin gels containing 10^−7 ^M~10^−5 ^M of ALN resulted in enhanced cell proliferation and osteoblastic differentiation of embedded hMSCs.

We used a rabbit calvarial defect model to evaluate the capacity of fibrin/ALN gels to regenerate bone after injury. Several studies report enhanced bone formation following local delivery of ALN [33, 34]. Toker et al. reported that an autogenous bone graft with ALN enhanced new bone formation in a rat calvarial defect model [33]. Komatsu et al. found that local application of ALN promoted bone formation in a rat tooth replantation model, and the bone formation effects may have occurred through endocytic incorporation of ALN and subsequent inhibition of protein prenylation [34]. For our in vivo study, we used fibrin gel prepared with 10^−6 ^M ALN because of its superior cellular cytocompatibility. After implantation of the fibrin/ALN gel into the calvarial defect, newly formed bone, in the form of an island, was visible in the central defect area. Furthermore, the newly formed bone increased in the fibrin/ALN gel-implanted group compared to that in the fibrin gel group. Although the precise biological mechanism of fibrin/ALN gel involved in bone formation was not revealed in this study, our findings suggest that improved bone formation occurred via stimulation of proliferation and differentiation of bone-forming cells by ALN released from the fibrin/ALN gel.

## 5. Conclusions

We prepared fibrin/ALN gels for ALN local delivery to improve bone regeneration. The fibrin/ALN gel provided a suitable environment for hMSC proliferation and differentiation in vitro. Furthermore, fibrin/ALN gels enhanced new bone formation in a rabbit calvarial defect in vivo. This study highlighted the potential of fibrin/ALN gel for drug delivery and sustained release for bone tissue regeneration applications. Further studies should focus on enhancing bone defect regeneration by varying the fibrinogen and thrombin concentrations in fibrin/ALN gels.
